# Comprehensive Characterization of Tissues Derived from Animals at Different Regenerative Stages: A Comparative Analysis between Fetal and Adult Mouse Skin

**DOI:** 10.3390/cells12091215

**Published:** 2023-04-22

**Authors:** Valentina Castillo, Pamela Díaz-Astudillo, Rocío Corrales-Orovio, Sebastián San Martín, José Tomás Egaña

**Affiliations:** 1 Institute for Biological and Medical Engineering, Schools of Engineering, Biological Sciences, and Medicine, Pontificia Universidad Católica de Chile, Santiago 7820436, Chile; 2 Biomedical Research Center, School of Medicine, Universidad de Valparaiso, Valparaiso 2540064, Chile; 3 Division of Hand, Plastic and Aesthetic Surgery, University Hospital, LMU Munich, 81377 Munich, Germany

**Keywords:** tissue regeneration, tissue repair, skin, extracellular matrix

## Abstract

Tissue regeneration capabilities vary significantly throughout an organism’s lifespan. For example, mammals can fully regenerate until they reach specific developmental stages, after which they can only repair the tissue without restoring its original architecture and function. The high regenerative potential of fetal stages has been attributed to various factors, such as stem cells, the immune system, specific growth factors, and the presence of extracellular matrix molecules upon damage. To better understand the local differences between regenerative and reparative tissues, we conducted a comparative analysis of skin derived from mice at regenerative and reparative stages. Our findings show that both types of skin differ in their molecular composition, structure, and functionality. We observed a significant increase in cellular density, nucleic acid content, neutral lipid density, Collagen III, and glycosaminoglycans in regenerative skin compared with reparative skin. Additionally, regenerative skin had significantly higher porosity, metabolic activity, water absorption capacity, and elasticity than reparative skin. Finally, our results also revealed significant differences in lipid distribution, extracellular matrix pore size, and proteoglycans between the two groups. This study provides comprehensive data on the molecular and structural clues that enable full tissue regeneration in fetal stages, which could aid in developing new biomaterials and strategies for tissue engineering and regeneration.

## 1. Introduction

Tissue regeneration capacities vary significantly between different organisms. For instance, some invertebrates, such as planarians, hydras, and starfish, are well known for regenerating and fully restoring missing parts of their bodies [1,2]. This capacity is not limited to “lower” organisms, as some vertebrates, such as axolotls, newts, and zebrafish, also have the potential to regenerate full limbs, appendices, hearts, brains, muscles, and skin [3]. Interestingly, the regenerative capacity of these organisms remains throughout their lifetimes and is not affected by aging [4]. However, in contrast to these species, mammals can only regenerate at early stages and lose this capacity over time. For example, fetal scarless wound repair is characterized by restoring the original tissue architecture and function, and the transition from a scarless to scarring phenotype occurs in the third trimester of pregnancy in humans and around embryonic day 18 (E18) in mice [5,6,7,8]. However, later studies demonstrated that scarless wound healing occurs in the mouse dorsum until stage E16.5, and fetuses at E18.5 suffer scarring [8].

Compared with adults, fetal wound repair is characterized by rapid epithelialization, connective tissue deposition, and fibroblast migration [8]. Furthermore, the correlation between tissue regeneration impairments and aging has been broadly described in humans [9,10,11].

Scarless wound healing has been well documented in animals such as axolotls and zebrafish [12,13]. It has also been studied extensively in human and murine models during fetal stages [14,15]. The high regenerative potential observed in fetuses is attributed to several local and systemic factors, including the immune system, which plays a central role in orchestrating the tissue-healing process. Until the second trimester, fetal skin contains lower levels of immune cells and inflammatory cytokines, making immune deficiency a critical underlying mechanism of scarless healing [16,17]. In fact, the wound microenvironment at such stages is associated with a lack of inflammatory molecules. On the other hand, some anti-inflammatory cytokines such as interleukin (IL)-10 are highly expressed in mid-gestation human fetal wounds, resulting in a permissive environment for scarless wound repair, but are absent in postnatal human skin [18].

In addition to immune cells, mesenchymal stem cells (MSCs) play an essential role in aiding all phases of the wound-healing process [19]. They decrease inflammation and immune function while increasing the proliferation and differentiation potential of epidermal cells, fibroblasts, and endothelial cells [19]. Moreover, in aged skin, MSCs suffer a significant loss in number and functionality, resulting in a decline in tissue maintenance and regenerative capacity in the long term [20,21]. However, adult MSC cells can be intrinsically “reprogrammed” to adopt a more youthful phenotype, enhancing tissue repair in older organisms [22,23]. Fibroblasts are also highly relevant local cells as they regulate the wound healing process at different levels., including the secretion of growth factors (GF) and the synthesis and remodeling of the extracellular matrix (ECM). Fetal and adult fibroblasts display differences in the synthesis of collagen, hyaluronic acid, and other extracellular matrix components. For example, fetal fibroblasts synthesize more Collagen III and have more surface receptors for hyaluronic acid, enhancing migration. Additionally, fetal wound healing is characterized by the absence or low levels of myofibroblasts, a type of fibroblast with the ability to contract, which correlates with scarring [5,24,25].

Indeed, distinct fibroblast lineages determine dermal architecture in skin development and repair, showing the differential expression of markers during prenatal [26,27] and postnatal development [28].

Growth factors play a critical role in wound healing, as they coordinate several cellular and molecular processes. However, only a few have shown an anti-fibrotic effect, being differentially regulated in the skin during regenerative and reparative stages. For example, previous studies have shown that the increased expression of transforming growth factor-beta 1 (TGF-β1), TGF-β2, platelet-derived growth factor (PDGF), fibroblast growth factor (FGF), and vascular endothelial growth factor (VEGF) can enhance fibrosis and scar formation in adult organisms [5,29,30,31]. On the other hand, TGF-β3 and heparin-binding epidermal growth factor (HB-EGF) have been found to promote scarless wound healing, being increased during fetal wound healing [5,32].

ECM provides a dynamic scaffold for cell migration, adhesion, proliferation, differentiation, and maturation; therefore, it is critical for wound repair. Within this context, differences between fetal and adult ECM have also been described. For instance, fetal-skin ECM contains more hyaluronic acid than adult skin, and fetal fibroblasts express more hyaluronic acid receptors [33,34]. In scarless fetal wounds, the hyaluronic acid content of the ECM increases more rapidly than in adult wounds [35]; for this reason, several studies have proposed a key role for hyaluronic acid in scarless healing [35,36,37]. Additionally, the ratio between tissue-derived inhibitors and the proteins in charge of ECM remodeling, or matrix metalloproteinases (MMPs), is lower in adult wound healing, favoring collagen accumulation and, therefore, scarring [5,38].

The studies mentioned earlier provide cellular and molecular data about the factors that regulate tissue repair and regeneration, with a primary focus on the differences between fetal and adult tissues. Although this phenomenon is well documented, to the best of our knowledge, no comprehensive integrative studies have been conducted to compare tissues obtained from animals at different stages of regeneration, such as embryos and adults. Therefore, we conducted a comprehensive analysis comparing skin samples from mice at regenerative (E16.5) and reparative (6–8 weeks) stages.

## 2. Materials and Methods

### 2.1. Mice Breeding

The use of animals in this study followed the National Institutes of Health Guide for the Care and Use of Laboratory Animals. The Institutional Animal Care and Use Committee of Pontificia Universidad Católica of Chile approved the protocol (CEC-CAA 190613020). Female C57BL/6 mice (6–8 weeks) and fetuses at the E16.5 stage were used. Female mice were selected based solely on their availability in our local animal facility during the experiments.

The fetal stage was determined according to our breeding facility’s protocols. Briefly, female and male mice were placed together in a box for one day to mate. The presence of the copulatory plug confirmed conception and denoted day 0.5 of gestation. After breeding, the female mice were individually housed, and weight gain was used as an indicator of a successful pregnancy for those selected as candidates. Euthanasia was performed via carbon dioxide inhalation, and every effort was made to minimize suffering.

### 2.2. Skin Tissue Dissection and Biopsy Sampling

Following euthanasia, the back of each adult mouse was shaved using a machine and then treated with a depilatory cream for 2 min. After thorough washing with water, skin was carefully dissected from the back of the animal using tweezers and thin surgical scissors. For fetus retrieval, a cesarean section was performed, and skin was obtained from the back of the fetus using a scalpel and fine tweezers.

The removed skin was washed once for 5 min with saline and then fixed in 4% paraformaldehyde (PFA) or stored in saline at 4 °C until the next day for further analysis.

Unless otherwise specified, all experiments were performed using the entire back skin harvested from fetuses and 8 mm diameter biopsy samples obtained from adult skin. In this work, fetal and adult skin samples are referred to as regenerative and reparative samples, respectively.

### 2.3. Macroscopic Visualization

Regenerative and reparative fresh samples were visualized using a stereo microscope (LeicaS6D, Leica, Wetzlar, Germany) coupled with a digital camera (MS60, Mshot, Guangzhou, China).

### 2.4. Molecular Analysis

#### 2.4.1. Nuclear Visualization and DNA Quantification

Regenerative and reparative skin samples (N = 3) were fixed in 4% PFA for 24 h at 4 °C and then dehydrated in 30% sucrose for 24 h at 4 °C. Cryosections of 5 µm were prepared from the skin samples, washed in phosphate-buffered saline (PBS), and then incubated in Hoescht 3342 at a concentration of 1 µg/mL (Thermo, Waltham, MA, USA) for 20 min in PBS, protected from light. The sections were examined and photo-documented using standard fluorescent microscopy (DM500, Leica, Wetzlar, Germany) coupled with a digital camera (MS60, Mshot, Guangzhou, China).

Nuclear density quantification was performed on the Hoescht-stained images, calculating the percentage of area covered by the fluorescence signal using the Fiji software [39]. A distribution analysis of nuclear signal percentage across the *X*-axis and *Y*-axis of each photo was also performed for the regenerative and reparative skin using the same software [39]. For DNA quantification, the skin samples were freeze-dried for 24 h at 0.05 Pa and −60 °C (SJIA-10N, SJ Lab, Ningbo, China), followed by DNA extraction, performed according to manufacturer instructions (DNA Isolation Kit for Cells and Tissues, Roche, Basel, Switzerland). The total DNA content was quantified using an Epoch2 Microplate Spectrophotometer (BioTek Instruments, Winooski, VT, USA) and normalized with the dry weight of each sample.

#### 2.4.2. Lipid Visualization and Quantification

Regenerative and reparative skin samples (N = 3) were fixed in 4% PFA for 24 h at 4 °C, followed by dehydration in 30% sucrose for 24 h at 4 °C. The samples were then cut into 5 µm thick sections and washed in PBS. The sections were incubated with BODIPY 493/503 Lipid Probes (1 µg/mL) plus Hoescht 3342 (1 µg/mL) for 20 min in PBS, protected from light. The sections were examined and photographed using standard fluorescent microscopy (DM500, Leica, Wetzlar, Germany) coupled with a digital camera (MS60, Mshot, Guangzhou, China).

Neutral lipid density was quantified from the area covered by the BODIPY stain using the Fiji software [39], and the total area (100%) was considered the area covered by nuclei (Hoechst stain). A distribution analysis of the lipid signal percentage across the *Y*-axis (upper to lower) and *X*-axis (left to right) of each photo was also performed using the same software [39]. Colocalization with the Hoechst stain was quantified using the BIOP JACoP Plugin of Fiji [39].

### 2.5. Histological Stains and Immunohistochemistry

Isolated skin samples (N = 9) were fixed in 4% PFA or methacarn, dehydrated in ethanol, and embedded in Paraplast at 60 °C (Leica Biosystems, Wetzlar, Germany). Sections of 5 μm in thickness were cut and adhered to glass slides using 0.1% poly-L-Lysine (Sigma-Aldrich, St. Louis, MO, USA) and dried at room temperature.

Histochemical stains were performed for the detection of ECM molecules such as fibrillar collagen, elastic fibers, and carboxylated glycosaminoglycans (cGAGs) using Masson’s trichromic stain, Herovici (aniline blue and acid fuchsin), orcein, and Alcian Blue 8 GX at pH 2.5, respectively. Image analysis and quantification were performed using the Color Deconvolution tool, followed by Fiji software threshold adjustment [39]. Densitometric analysis was performed using the mean gray value, which is the sum of the gray values of all the selected pixels divided by the number of square pixels.

Immunohistochemistry was performed using a previously established protocol [40]. Briefly, sections were deparaffinized and rehydrated; when needed, antigen retrieval was performed by incubating slides at 95 °C for 30 min in 10 mM of citrate buffer, pH 6. Then, endogenous peroxidases were blocked with 3% hydrogen peroxide in PBS. Samples were then incubated with the following antibodies in PBS containing 0.3% (*v*/*v*) Tween 20 (Winkler, Santiago, Chile) overnight at 4 °C: rabbit monoclonal Ki67 (MA5-14520, Invitrogen, MA, USA); rabbit polyclonal anti-Collagen XVIII alpha chain (SAB4503212, Sigma-Aldrich, St. Louis, MO, USA), diluted 1:50; rabbit monoclonal primary anti-Tenascin C (AB108930, Abcam, Cambridge, UK), diluted 1:200; rabbit polyclonal anti-Versican (Abx100119) diluted 1:150; rabbit polyclonal anti-Perlecan (Abx176806), diluted 1:50; rabbit polyclonal anti-Agrin (Abx129847), diluted 1:100; and rabbit polyclonal anti-Fibromodulin (Abx004875), diluted 1:200. The last four antibodies were purchased from Abbexa (Cambridge, UK). Nonspecific staining was blocked via 30 min of immersion in Cas-Block solution (Thermo, Waltham, MA, USA) and goat serum (Gibco, Carlsbad, MA, USA). After extensive rinsing in PBS, all sections were incubated for 1 h at RT with HRP-conjugated goat anti-rabbit IgG (Sigma-Aldrich, St. Louis, MO, USA), 1:1000 in PBS. The peroxidase reaction was visualized using the NovaRED kit (Vector Laboratories, Newark, CA, USA). After immunostaining, sections were slightly stained with Harris hematoxylin (Merck, Branchburg, NJ, USA). For each immunohistochemical reaction, controls were performed by incubating the sections with PBS by omitting the primary antibody. Sections were examined using standard light microscopy (CX43, Olympus, Tokyo, Japan) coupled with a digital camera (4K Sony Ultra HD VC.3040, Euromex, Arnhem, The Netherlands).

### 2.6. Sulfated Glycosaminoglycans (sGAGs) Visualization and Quantification

Frozen samples of both regenerative and reparative skin (N = 9) underwent freeze-dried for 24 h at −60 °C and 0.05 Pa (SJIA-10N, SJ Lab, Ningbo, China). Quantitative analysis of sGAG content was performed using Blyscan Assay (Biocolor, Belfast, UK) following the supplier’s instructions. All samples were analyzed in duplicate and read at 656 nm in a spectrophotometer (GENESYS 10 uv, Thermo, Waltham, MA, USA). The final concentration of sGAGs was determined from a calibration curve built from the provided standards, with an r^2^ value of 0.99 or higher.

### 2.7. Scanning Electron Microscopy (SEM) and Structural Analysis

To analyze the samples from regenerative and reparative skin (N = 3), the freeze-drying process was applied for 24 h at 0.05 Pa and −60 °C (SJIA-10N, SJ Lab, Ningbo, China). The samples were then mounted, sputtered with gold, and imaged using 15 kV of accelerating voltage (TM3000, Hitachi, Tokyo, Japan). Three representative images of each sample (N = 3 per group) were selected for the analysis, and ten different pores per image were measured for pore diameter calculation, corresponding to ninety pores per group. Porosity was defined as the percentage of empty area for each sample, which was the mean of the three images each. Both pore size and porosity analyses were performed using the Fiji software [39], adjusting the threshold followed by the straight tool or threshold followed by the fraction area (%) calculator for pore size and porosity, respectively.

To calculate fiber thickness, at least one image of each sample (N = 3 per group) was used for the analysis, and ten different fibers per image were measured for the pore fiber thickness calculation. The analysis was performed by measuring the length with the manual straight tool of the Fiji software [39].

### 2.8. Functional Analysis

#### 2.8.1. Metabolic Activity

The metabolic activity of the skin was assessed using an Oxygraph^+^ System (Hansatech Instruments, King’s Lynn, UK). Freshly isolated samples obtained from regenerative and reparative skin (N = 3) were placed in the electrode chamber of the oxygraph and covered with 1 mL of saline. The concentration of dissolved oxygen was measured for 10 min, and the metabolic activity (oxygen consumption rate) was determined from the slope of the oxygen concentration curve. The values were then adjusted based on the dry weight of each sample.

#### 2.8.2. Water Absorption Capacity

To determine the percentage of water absorption, swelling studies were performed. Freshly isolated skin samples from regenerative and reparative (N = 3) samples were submerged in 1 mL of distilled water for 24 h and weighed before (*Ww*) and after (*Wd*) drying over clean filter paper. The ratio of swelling was determined using the following equation [41]:

$$\mathrm{Swelling}\;\mathrm{ratio}\;=\frac{Ww-Wd}{Wd}$$

#### 2.8.3. Mechanical Characterization

Mechanical properties of regenerative and reparative skin samples (N = 3) were studied by performing compression tests using a TA-XT plus texture analyzer (Stable Micro Systems, Godalming, UK). The tests were carried out using a 10 mm diameter cylindrical probe, and the samples were compressed until they reached 70% strain. The compressive Young modulus was determined by calculating the slope of the linear region of the stress–strain curve. To ensure complete contact with the probe geometry, larger biopsies from reparative skin (10 mm punches) were used for this analysis.

### 2.9. Statistical Analysis

The data are presented as mean values plus the standard error of the mean (SEM). Statistical significance was evaluated by performing an unpaired one-tailed *t*-test or one-way ANOVA Tukey’s multiple comparisons tests after the Shapiro–Wilk test for normal distribution. All experiments were carried out in at least three independent assays, and a *p*-value < 0.05 was considered statistically significant. The level of statistical significance was categorized as follows: * *p* < 0.05, ** *p* < 0.01, *** *p* < 0.001, and **** *p* < 0.0001 according to the GraphPad Prism software.

## 3. Results and Discussion

### 3.1. Macroscopic Analysis

**Figure 1:**
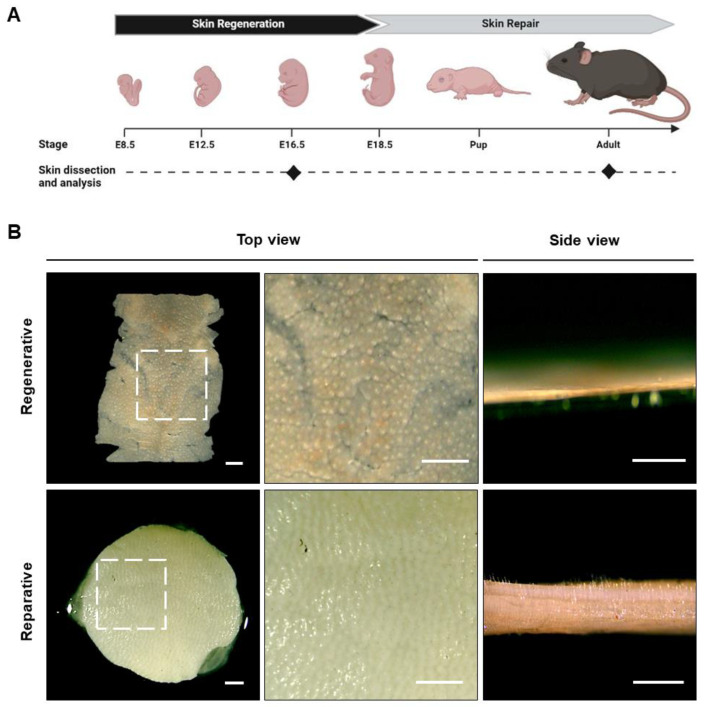
Skin biopsies derived from mice at the regenerative and reparative stages. (**A**) Schematic representation of the mouse stage used for skin dissection, created using Biorender (https://biorender.com, accessed on 28 January 2023). (**B**) Macroscopic appearance of regenerative and reparative skin. For animals at the regenerative stage, the entire skin sample from the back is shown, while an 8 mm diameter biopsy sample is shown for the reparative stage. The right panels show the magnified area, indicated by a dotted-line square in the left images. Scale bars represent 1 mm.

To evaluate differences between tissues at regenerative and reparative stages, as shown in Figure 1A, mouse skin was harvested from the fetus at E16.5 (the regenerative stage) and from adult mice of 6–8 weeks old (the reparative stage), and the macroscopic appearance was observed. As shown by the scale bar in Figure 1B, the skin from the reparative stage was ten times thicker than at the regenerative stage, with values of about 700 and 70 µm, respectively. Additionally, while no differences were observed in the epidermal thickness of both groups, a significantly thicker dermis was detected in the reparative skin (Figure S1A), which is reflected in a six-times-larger dermal-to-epidermal ratio (Figure S1B).

Although no direct correlation between animal size and skin thickness is observed in nature, since adult mice are about six times larger than embryos at the E.16.5 stage [40,41], a significant difference was expected. Hair follicle-like structures were observed in both samples (Figure 1B, top view magnification); however, only the reparative skin exhibited hairs over the epidermal layer (Figure 1B). At this point, it is important to mention that a drawback of this study is that adult skin was obtained only from female mice, and, therefore, further studies should validate this data for male skin. Indeed, previous studies have shown that adult male mice have thicker skin than their female counterparts, with the major contributor to these differences coming from a thicker dermal layer. In fact, the dermal layer was found to be almost three times thicker in male skin compared with female skin. However, according to the same study, the epidermis and hypodermis layers exhibited opposite behavior, being thicker in female mouse skin than in male mouse skin [40].

Although there are no reports indicating that sex-related differences in skin morphology affect cutaneous wound healing, some studies have suggested the presence of sexual dimorphism in wound healing that is associated with differential levels of sex steroid secretion [42]. For instance, it has been found that female mice have an advantage in the wound-healing process and angiogenesis [43], which is regulated by estrogen. This hormone is known to regulate the expression and/or pro-angiogenic activity of A2A adenosine receptors, likely involving the activation of ERα and ERβ receptors [43]. Therefore, given the sex-associated differences in regenerative potential, future analyses performed under injury conditions should include male and female mice.

### 3.2. Cellular and Molecular Features

**Figure 2:**
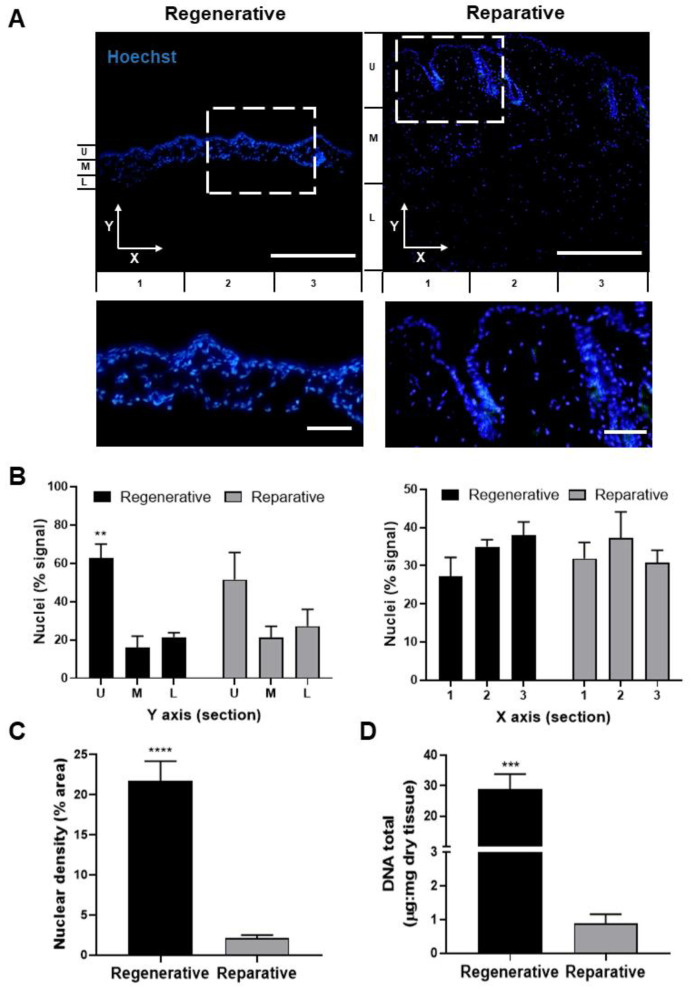
Nuclei distribution and the DNA content of skin at the regenerative and reparative stages. (**A**) Transversal tissue sections stained with Hoechst nuclear probe. (**B**) The distribution of nuclei in the *Y*-axis (left) and *X*-axis (right) sections is expressed as a relative percentage of the total signal. The bottom panels show the magnified area indicated by a dotted-line square in the upper images. (**C**) Nuclear density expressed as a percentage of the area covered by nuclei. (**D**) Spectrophotometric quantification of total dsDNA content from regenerative and reparative skin relative to its dry weight. Data are presented as mean + SEM (N = 3) and analyzed using an unpaired *t*-test or one-way ANOVA Tukey’s multiple comparisons tests. ** *p* < 0.01; *** *p* < 0.001; **** *p* < 0.0001. Scale bars represent 50 µm and 10 µm for lower and higher magnification, respectively.

To compare skin at the regenerative and reparative stages of mice, transverse skin sections were stained, and the cell content and distribution were evaluated (Figure 2A). Nuclear distribution across the *X-* and *Y*-axes of the images was found to be conserved between both groups. As described, the nuclear distribution across the *Y*-axis showed a standard and specific pattern with an increased signal in the epidermal or upper segment (U) compared with the medium (M) and lower (L) segments of the sample (Figure 2B, left). Even though a similar distribution pattern was observed in both tissues, significant differences were quantified only for the regenerative epidermis. In contrast, no significant differences were observed along the *X*-axis when comparing both groups (Figure 2B, right). Additionally, the nuclear density was significantly higher in regenerative than reparative skin, with an average of 21.7% and 2.1% of the areas covered by nuclei, respectively (Figure 2C).

Next, the DNA content of both skin samples was quantified and compared, showing significantly higher levels in the regenerative skin than the reparative skin samples, with an average of 28.8 µg/mg dry tissue and 0.89 µg/mg dry tissue, respectively (Figure 2D).

Despite the visible differences in Figure 1B, the same cellular distribution pattern was detected between the regenerative and reparative stages of the skin. However, the nuclear density and DNA content were significantly higher in the regenerative skin. The latter could be attributed to the increased cell proliferation rate that characterizes tissue growth during embryonic development [44], which, in contrast to adult tissue, has not yet reached maturity. Moreover, the higher cell density may also be due to the low ECM content described for regenerative skin [45].

Besides their well-described function as an essential component of cell membranes, energy storage [46], and the barrier function of the skin [47], the state of the art about lipids in cell biology has changed over the last few years. They are now also described as signaling molecules involved in immune responses, inflammation, and tissue homeostasis [48], which in turn also play a critical role in tissue regeneration [49].

**Figure 3:**
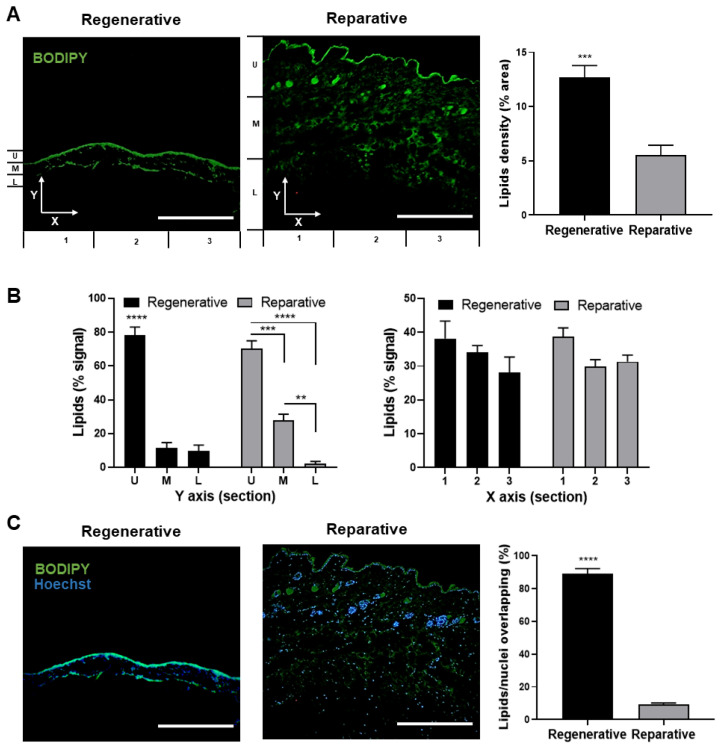
Lipid distribution of skin at regenerative and reparative stages. (**A**) Transversal skin sections stained with BODIPY 493/503 probe showing neutral lipid (left) and its density expressed as a relative percentage of the total area. (**B**) Distribution of lipids in the *Y*-axis (left) and *X*-axis (right) is expressed as a relative percentage of the total signal. (**C**) Skin sections co-stained with Hoechst and BODIPY 493/503 showing neutral lipid overlapping with nuclei (left) and its quantification (right). Data are presented as mean + SEM (N = 3) and analyzed using an unpaired *t*-test or one-way ANOVA Tukey’s multiple comparisons tests. ** *p* < 0.01; *** *p* < 0.001; **** *p* < 0.0001. Scale bars represent 50 µm.

Neutral lipids were stained and analyzed in skin samples from animals in the regenerative and reparative stages (Figure 3). The tissue area covered by neutral lipids was significantly different between groups, with an average of 12.7% in the case of regenerative skin, while it was only 5.5% in reparative skin (Figure 3A).

The distribution of neutral lipids across the Y- and X-axes of the images was found to be slightly different between the two groups. The lipid signals across the *Y*-axis showed a different distribution pattern for each group. In regenerative skin, there was an increased signal in the epidermal or upper segment (U) compared with the medium (M) and lower (L) sections (Figure 3B, left, black bars). In contrast, a “stairs pattern” with a high-to-low signal from the upper to lower sections was observed for the reparative skin (Figure 3B, left, gray bars). Furthermore, the data showed strong colocalization between neutral lipids and nuclear signals in the regenerative skin, with 89.3% of signals overlapping, while only a 9% overlap was observed in skin derived from the reparative stage (Figure 3C).

These results show that both regenerative and reparative skin have a lipid layer that coincides with the location of the stratum corneum. This structure is primarily composed of corneocytes, which are surrounded by an extracellular lipid matrix [50]. This lipid composition is not only important for the skin’s physical barrier function but also plays a role in skin permeability, which has been reported to be a marker of maturity, decreasing from the E16 gestational stage when the skin barrier is formed [51]. Interestingly, in fetal wound healing, increased permeability could allow for a better exchange of soluble factors between the regeneration tissue and the environment, including metabolic waste, in which accumulation has been shown to be a critical issue for repair and regeneration [52]. Furthermore, the results showed that adult skin has more lipids distributed throughout the dermis. This may be explained by the presence of dermal white adipose tissue, which is located adjacent to hair follicles and modulates their periodic growth and wound healing. The localization and size of dermal white adipose tissue may vary depending on the hair cycle stage and even housing conditions [53]. Additionally, this lipid distribution in adults could be associated with energy storage and thermal insulation, while the absence of lipids throughout the dermis of fetal skin could be explained by the fact that, in comparison with adults, at that stage, insulation and energy are obtained from the mother.

Among the wide variety of lipids, neutral lipids such as cholesterol esters, triglycerides, and wax are wildly known to be parts of cellular membranes and lipid droplets (LDs). The results here showed the high colocalization of lipids and nuclei in the regenerative skin, which could indicate the presence of nuclear LDs. Interestingly, such droplets have been linked to diverse cellular functions and play an important role as regulators of cellular metabolism [46,48]. However, to the best of our knowledge, the prevalence of nuclear LDs in regenerative tissues have not yet been reported. Therefore, their association with the regeneration process should be further studied in more detail. 

### 3.3. ECM Composition and Structure

The fetal ECM is a dynamic structure composed mainly of proteins and GAGs that undergoes a series of changes before reaching its adult phenotype. It is optimized to facilitate tissue growth, cellular migration, and proliferation. Thus, the fetal ECM could significantly contribute to providing the pro-regenerative microenvironment found in embryos [5]. Therefore, this study performed a detailed characterization of the ECM using several methods and techniques.

#### 3.3.1. Fibrous Proteins

**Figure 4:**
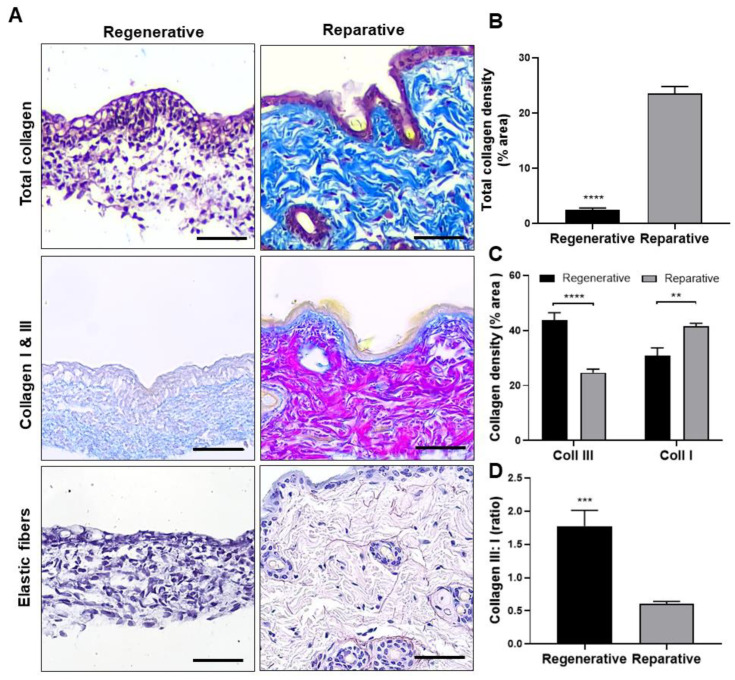
Fibrous protein distribution of skin at the regenerative and reparative stages. (**A**) Skin sections were stained with Masson’s trichrome for total collagen (upper), Herovici for Collagen I (pink stain) and III (blue stain) (middle), and orcein for elastic fiber visualization (lower). (**B**) Total collagen density expressed as a percentage of the area covered by total collagen. (**C**) Collagen I and III density expressed as a percentage of the area covered by each collagen type. (**D**) Collagen III:I ratio plot. Results are shown as mean + SEM (N = 9) and analyzed using an unpaired *t*-test or one-way ANOVA Tukey’s multiple comparisons tests. ** *p* < 0.01; *** *p* < 0.001; **** *p* < 0.0001. Scale bars represent 50 µm.

Collagen and elastic fibers are the primary structural proteins responsible for the mechanical properties of the ECM. Therefore, a histological analysis was conducted to compare regenerative and reparative skin samples (Figure 4A), revealing significant differences in the number and distribution of these components. Masson’s trichrome stain (Figure 4A, upper) showed that collagen fibers (blue staining) were mainly distributed in the dermal layer in both groups, exhibiting significantly lower density in the regenerative skin, with a 9.5-fold increase observed in reparative skin samples (Figure 4B). Herovici staining was performed to differentiate between Collagen I and III (Figure 4A, middle), revealing an inverted distribution in both groups. In the reparative skin, there was a predominance of Collagen I (pink staining) in the reticular dermal layer, while Collagen III (blue staining) was mainly found in the papillary dermis. Quantitative analysis showed that Collagen I was significantly less abundant in the regenerative skin than the reparative skin, while Collagen III was significantly higher (Figure 4C). As a result, the Collagen-III-to-I ratio was significantly higher in the regenerative skin than in the reparative skin (Figure 4D). Finally, orcein staining (Figure 4A, lower) showed that the reparative skin exhibited abundant levels of elastic fibers compared with the regenerative skin, where only a few such fibers were found; this is the reason why a quantitative comparison was not performed.

Overall, the histological analysis revealed significant differences between the ECMs of the regenerative and reparative skin, which is consistent with previous reports describing collagen deposition in fetal and adult skin [45,54]. Collagen is the major component of the ECM, and during the wound-healing process, it modulates cell proliferation and migration and is essential in the wound contraction process [55]. The results presented here show a shift from Collagen III to I Collagen in fetal development, which correlates with the previously reported transition from scarless regeneration to scar formation [56]. Furthermore, Collagen III has also been found to be an essential contributor to tissue regeneration by regulating myofibroblast differentiation and scar formation in cutaneous wound healing [57]. Interestingly, it has also been found that Collagen III is first expressed during wound healing in adults and later replaced by Collagen I produced by mature fibroblasts. Therefore, the repair process in adult tissue somehow follows a similar pattern to fetal development, where a higher amount of Collagen III is present [58], providing insights into the potential role of Collagen III in the regeneration of skin tissue. In addition to collagen, elastic fibers are another essential fibrous protein to the ECM. Previous reports have shown that elastin is produced during fetal development [44], and tropoelastin in fetal mouse skin has been found as early as embryonic day 10 [59]. However, the findings of this study suggest that, at gestational stage E16.5, tropoelastin does not yet mature into elastin fibers, as shown by the absence of elastic fibers in the orcein stain. Since the composition and architecture of the ECM play a significant role in cell function, a lower number of elastic fibers in regenerative skin could be important for scarless healing by modulating tissue elasticity or the expression and adhesion of other proteins. Previous reports have shown scarless wound healing is achieved only between early- and mid-gestation, in a developmental period before a fully functional elastic fiber network has formed [44]. Tropoelastin and elastin peptides modulate cellular behavior in various cell types to elicit biological responses such as monocyte chemotaxis, fibroblast migration, proliferation, and protease production [60].

#### 3.3.2. Glycosaminoglycans

**Figure 5:**
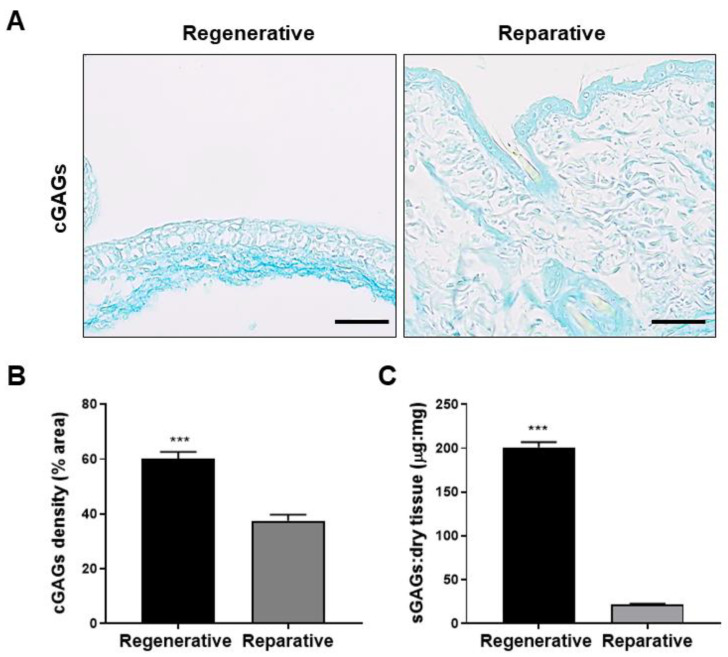
Glycosaminoglycan (GAG) content of skin at the regenerative and reparative stages. (**A**) Carboxylated glycosaminoglycans (cGAGs) stained with Alcian blue, pH 2.5. (**B**) cGAG density expressed as a percentage of the area covered by cGAGs and (**C**) sGAGs levels expressed as µg per mg of dry weight tissue. Results are shown as mean + SEM (N = 9) and analyzed using an unpaired *t*-test. *** *p* < 0.001. Scale bars represent 100 µm.

Furthermore, the content and distribution of GAGs in both skin samples were also studied and compared (Figure 5). GAGs were categorized into carboxylated GAGs (cGAGs) and sulfated GAGs (sGAGs) based on their secondary group modifications. The results revealed that cGAGs were predominantly present in the dermis of the regenerative skin, whereas an epidermal distribution was observed in the reparative skin (Figure 5A). Additionally, densitometry analysis showed a 1.5-fold higher density of cGAGs in the regenerative skin compared with the reparative skin (Figure 5B). Moreover, sGAGs were quantified and demonstrated 10-fold higher content in regenerative skin compared with reparative skin samples (Figure 5C).

Of all the cGAGs, hyaluronic acid has been found to be a critical molecule in wound healing, as it promotes cell migration and proliferation in wounds [61,62]. Moreover, higher hyaluronic acid levels have been detected in fetal wound fluid compared to adult wounds [34]. Overall, the findings described above indicate that uninjured regenerative skin exhibits higher levels of GAGs compared with reparative skin.

#### 3.3.3. Ultrastructural Analysis

**Figure 6:**
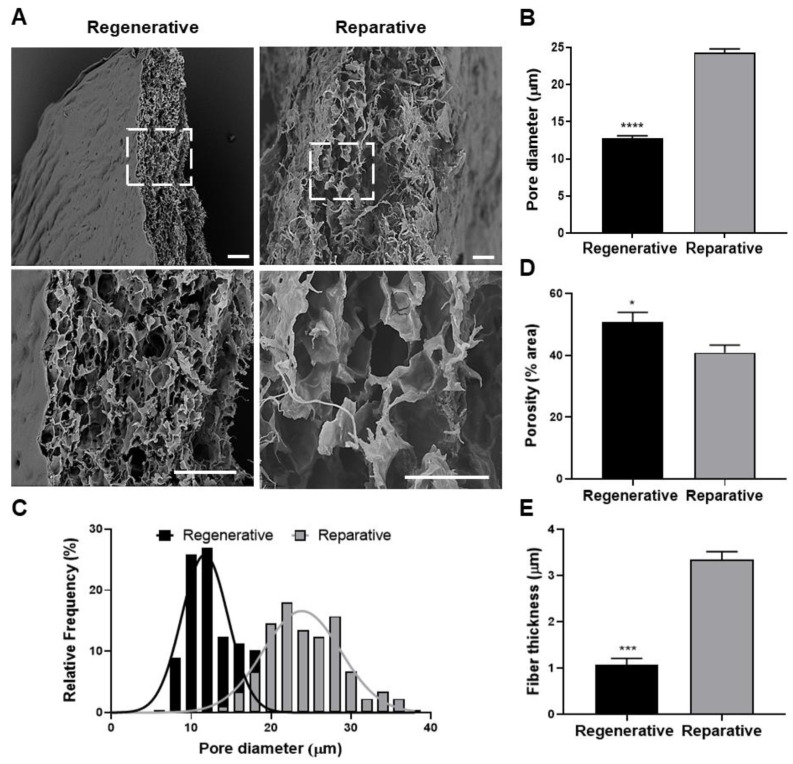
Ultrastructural comparison of skin at the regenerative and reparative stages. (**A**) Scanning electron microscopy. The bottom panels show the magnified area indicated by a dotted line square in the upper images. (**B**) Pore diameter of skin at the regenerative and reparative stages. (**C**) Pore diameter distribution shown as the relative frequency. (**D**) Porosity shown as a percentage of the empty area. (**E**) Fiber thickness of the samples. Data are shown as mean + SEM (N = 3) and analyzed using an unpaired *t*-test. * *p* < 0.05; *** *p* < 0.001; **** *p* < 0.0001. Scale bars represent 50 µm.

SEM analysis was conducted to gain further insights into the ultrastructural characteristics of the samples. As depicted in Figure 6A, both tissues exhibited a highly porous structure with interconnected pores in a random fibrillary matrix. However, pore diameter analysis revealed significant differences between the groups. Specifically, the regenerative skin exhibited a significantly smaller mean pore diameter of 12.8 µm compared with the reparative skin, with a mean pore diameter of 24.3 µm (Figure 6B).

Furthermore, the distribution of pore diameter between groups was also assessed. The regenerative skin displayed smaller pores with a narrowed distribution, ranging from 8 to 18 µm in diameter, where more than 50% of the pores fell within the 10–12 µm range. In contrast, reparative skin exhibited diameters ranging from 14 to 36 µm, with most of the pores (74%) distributed between 20 and 28 µm (Figure 6C). The Shapiro–Wilk test indicated that only the pore diameter distribution in reparative skin followed a normal distribution. However, when the pore diameter distribution was fitted with a Gaussian distribution, both groups displayed a similar fit with coefficients of determination (r^2^) of 0.88 and 0.91 for the regenerative and reparative tissues, respectively.

Moreover, slight but significant differences in porosity were observed between the two groups, with the regenerative skin exhibiting higher porosity (50.8%) compared with the reparative skin (40.8%) (Figure 6D). Finally, the fiber thickness of the regenerative skin was significantly lower (a mean fiber thickness of 1 µm) compared with the reparative skin (a mean fiber thickness of 3.3 µm) (Figure 6E).

Cell migration and proliferation are essential for tissue formation and regeneration, and it has been widely observed that higher scaffold porosity is associated with improved cell proliferation and migration [63,64]. Therefore, increased porosity in regenerative skin could be an additional crucial factor in scarless regeneration, allowing cells to migrate more quickly to the damaged site in cases of tissue injury. This characteristic, along with the higher cell content shown in Figure 2, may also play a key role in the regenerative process, as it promotes shorter and easier cell migration, necessary for tissue regeneration.

Additionally, highly porous networks have been found to facilitate angiogenesis, nutrient diffusion, and waste product removal [65], all of which are essential for rapid tissue regeneration. Finally, this analysis demonstrated that the regenerative skin presented smaller pores and thinner fibers than the reparative skin, which could be associated with the differential abundance of collagen types and elastic fibers observed in the regenerative and reparative stages. Indeed, Collagen III fibrils are more abundant in fetal skin and have been found to be thinner than type I fibrils [66].

#### 3.3.4. Proteoglycans and Glycoproteins

**Figure 7:**
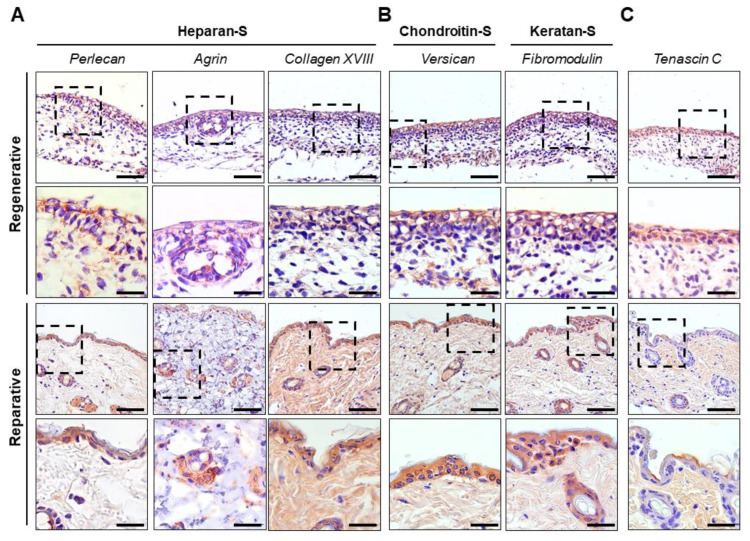
Immunohistochemistry of proteoglycans and glycoproteins of skin at the regenerative and reparative stages. (**A**) Heparan sulfates: Perlecan, Agrin and Collagen XVIII. (**B**) Chondroitin sulfate:Versican, and keratan sulfate:,Fibromodulin. (**C**) Glycoprotein Tenascin C. Dotted-line squares in images indicate magnified areas shown below. Scale bars represent 50 µm.

Immune-stained sections were qualitatively analyzed to further characterize the presence and distribution of proteoglycans and glycoproteins in the skin at the regenerative and reparative stages (Figure 7). Heparan sulfates are known to bind to several proteins present on the cell surface and ECM, as well as soluble growth factors and cytokines, modulating various cellular processes such as proliferation, adhesion, and migration [67]. Therefore, the presence and distribution of three heparan sulfate proteoglycans (Perlecan, Agrin, and Collagen XVIII) were studied (Figure 7A).

The Perlecan distribution showed a granulated pattern only in the regenerative skin, particularly in the pericellular space of the epidermis and sebaceous glands. In contrast, the reparative skin did not show the dotted pattern observed in the regenerative stage, but the distribution was conserved in the epidermis, sebaceous glands, and sebaceous ducts. Perlecan has been reported to contain Endorepellin in its sequence, an inhibitor of angiogenesis, and it also contains binding domains for FGF, PDGF, VEGF, and bone morphogenetic protein [67]. These interactions promote embryonic cellular proliferation, differentiation, and tissue development [67]. The expression pattern in the adult suggests its relation to the hair follicle cycle, while the pattern observed in regenerative skin suggests a role in regulating tissue development through its storage as granules and subsequent release in epithelial cells.

Agrin showed a granule-like stain on the cells, with a more pronounced pericellular epidermal distribution. This granulated pattern was conserved in the epidermis and the sebaceous glands in the reparative skin. Agrin is associated with long-term osteochondral and heart regeneration [37,68] and, more recently, with improved mechanoreception in skin wound healing, showing a similar distribution as reported herein [69].

On the other hand, Collagen XVIII showed a lower staining and heterogenous distribution in the regenerative skin. In contrast, at the reparative stages, it showed increased deposition in the dermal layer, while maintaining a ubiquitous expression in the cells of the epidermis and sebaceous glands (Figure 7A). This result is interesting because increased Collagen XVIII deposition in reparative skin could contribute to scarring. After all, the Collagen XVIII C-terminal fragment, also known as endostatin, is a well-known tumor growth and angiogenesis suppressor that inhibits endothelial cell proliferation and migration [70]. Additionally, mice lacking Collagen XVIII have shown faster wound healing with a loss of tissue organization [70]. In contrast, its overexpression has been associated with delayed wound healing and a loss of integrity due to epidermal detachment [70]. Moreover, Collagen XVIII is one of the molecules deposited early in mouse wound healing re-epithelization, and it has been suggested that it serves as an anchor for Collagen IV [71].

Because versican and fibromodulin have been suggested to be essential to scarless wound healing [72,73,74], their expression in the regenerative and reparative skins was also evaluated. While versican has been found to be highly expressed in human fetal skin compared with adult human skin [74], fibromodulin has been found in higher levels in fetal wounds [75], demonstrating that it is essential for fetal-type scarless cutaneous wound healing through the inactivation of TGF-β1, a profibrotic factor [72,75].

In this study, proteoglycans exhibited a similar staining pattern in regenerative and reparative skin, with a higher presence in the epidermal layer at both stages and in sebaceous gland cells during the reparative stage (Figure 7B). In contrast, staining on the dermis was only observed in the deep dermal layer of the regenerative tissue. These results are consistent with previous studies that have shown the expression of versican during regenerative skin development and its association with follicle maturation and cycling in adults [74,76].

Tenascin C is a well-described molecule involved in tissue remodeling [77] and has been shown to be upregulated in scarless fetal wounds [29,78]. Although much research has reported its role in injuries, there are limited data available on its role in non-injured regenerative and reparative tissues. As shown in Figure 7C, tenascin C was more widely distributed in the dermis and some points of the epidermal regions during the reparative stage compared with the regenerative stage, where it was primarily distributed in the epidermal region. This distribution is in line with previous studies demonstrating that tenascin C expression decreases with aging not only in the skin but also in other tissues [77]. Moreover, the study showed that tenascin C was ubiquitously expressed in regenerative skin, with a higher abundance in the epidermal layer and scarce distribution at the apical surface of epithelial cells. This ubiquitous distribution may be related to fetal tissue remodeling [79].

### 3.4. Functional Characterization

**Figure 8:**
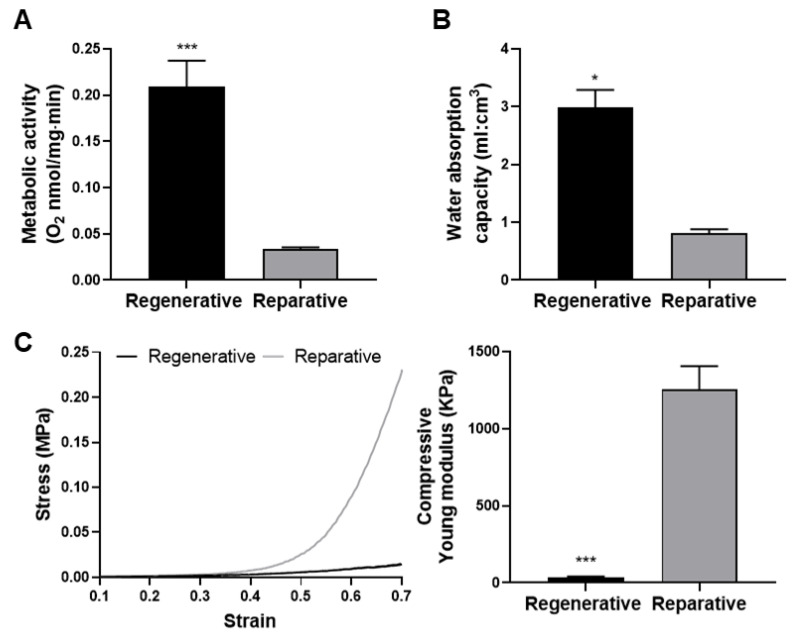
Metabolic and functional comparison of skin at the regenerative and reparative stages. (**A**) Metabolic activity of skin samples expressed as oxygen consumption normalized by its dry weight. (**B**) Water absorption capability of tissues relative to their volume. (**C**) Representative compressive stress–strain curves (left) and quantification of the Young modulus (right). Results are shown as mean + SEM (N = 3) and analyzed using an unpaired *t*-test. * *p* < 0.05; *** *p* < 0.001.

To compare the metabolic activity of skin derived from the regenerative and reparative mouse stages, oxygen consumption from freshly isolated samples was quantified via oxygraphy. The results showed significantly higher levels of consumption in the regenerative skin compared to the reparative skin (Figure 8A). This result correlates with the higher cell density described herein for regenerative skin and previous reports showing an increased cell proliferation rate during embryonic development [44]. Additionally, the water absorption capacity of tissues in both samples was significantly higher in the regenerative skin, which absorbed three times its own volume (Figure 8B). These results could be associated with the higher GAG content described above in the regenerative skin. As GAGs are highly hydrophilic, it is not surprising that the regenerative skin presented a higher water absorption capacity than the reparative skin. This property could be of high importance for optimal nutrient uptake as well as waste product removal, which is expected to be increased in highly metabolic tissues such as regenerative skin compared with reparative skin. Indeed, the swelling capacity is an important factor for tissue engineering scaffolds because it is essential for the absorption of body fluid and the transfer of cell nutrients and metabolites [41].

To obtain information about the mechanical properties of both tissues, stress–strain curves were acquired from a compressive deformation test (Figure 8C, left). The results showed that the Young modulus was significantly lower for the regenerative skin, exhibiting about 30-fold less stiffness than the reparative tissues (Figure 8C, right). This result is consistent with the results described above in this work, as the composition and architecture of the ECM determine the biomechanical features of tissues, and an increased number and thickness of collagen and elastic fibers were observed in the reparative skin. The first portion of the stress–strain curve has been found to be dominated by elastic fibers [80], showing a higher resistance in reparative skin. Moreover, the linear phase of the curve has been previously attributed to the collagen network’s resistance to deformation. Thus, the results found in the reparative and regenerative skin are positively associated with the total collagen and collagen ratio from the histological analyses [80], showing increased levels in the reparative stage compared with the regenerative stage.

Interestingly, these results agree with several studies describing the importance of tissue stiffness in cell behavior and tissue remodeling, where cell proliferation, migration, and differentiation are significantly impaired as ECM stiffness increases [81,82]. The stiffness differences observed between the compared tissues could contribute to the regenerative abilities observed in the regenerative skin by promoting a microenvironment conducive to cell motility and proliferation.

## 4. Conclusions

Early-gestation fetal skin possesses a unique ability to heal wounds without scarring. Because tissue regeneration is a complex multifactorial process, a comprehensive characterization is required to provide cellular and molecular clues regarding the factors that govern full tissue regeneration during fetal stages. Therefore, this study performed a detailed comparative analysis between skin derived from mice at the regenerative (E16.5) and reparative (6–8 weeks) stages.

The study found that regenerative and reparative skin exhibit differences at the molecular, structural, and functional levels, with the regenerative skin showing a significant increase in cellular density, nucleic acid content, neutral lipids density, Collagen III, and GAG content compared with reparative skin. The results also showed that lipid distribution, ECM pore size, and proteoglycans significantly differed between both groups. Moreover, the regenerative skin demonstrated significantly higher porosity, metabolic activity, water absorption capacity, and elasticity than the reparative skin.

However, further molecular and structural analyses must be performed to fully understand these features. For instance, characterizing the ECM progression during the development of mice could contribute to the knowledge base. Additionally, a quantitative analysis of ECM composition (e.g., mass spectrometry) could be performed to obtain an accurate comparison. A deep characterization of cell types and their abundance could also provide more insights. Finally, these results should be reproduced in human tissue to validate their clinical relevance.

It is worth mentioning that this work focuses on basal tissue conditions, which could be deeply altered after injury, where other factors may become protagonists. Nevertheless, this study provides knowledge regarding the composition and structure of native mouse skin, which could be directly related to mouse healing capacities. This provides critical scientific data and novel insights for developing advanced scaffolds for tissue engineering and regeneration. These scaffolds could be designed to mimic the composition, structure, and/or biomechanical properties of regenerative tissue, such as tissue containing a low amount of Collagen I, and a high amount of Collagen III and GAGs, together with high porosity and elasticity.

## Data Availability

The datasets generated for this study are available upon request from the corresponding author.

## Supplementary Materials

The following supporting information can be downloaded at https://www.mdpi.com/article/10.3390/cells12091215/s1: Figure S1: Layer thickness of regenerative and reparative skin. (A) Epidermal and dermal thickness quantified from H&E staining. (B) Ratio between dermal and epidermal thickness. Results are shown as mean + SEM (N = 4; 10 different measurements per sample) and analyzed using the Mann–Whitney and mixed model comparison test and the Tukey post-test. *p* < 0.0001. Figure S2: Cell proliferation. Ki67 staining was performed to visualize mitotic cells, showing positive cells in the regenerative skin (red arrowheads) as well as mouse spleen (positive control). In contrast, none were detected in the reparative skin. Scale bar represents 50 µm.
