# Supplementary material for: Comprehensive Characterization of Tissues Derived from Animals at Different Regenerative Stages: A Comparative Analysis between Fetal and Adult Mouse Skin
Source: Cells. 2023 Apr 22;12(9):1215. doi: 10.3390/cells12091215 (PMC10177150; doi:10.3390/cells12091215)
Supplement: Supplementary file 1 [file cells-12-01215-s001.zip › cells-2249405-supplementary.pdf]

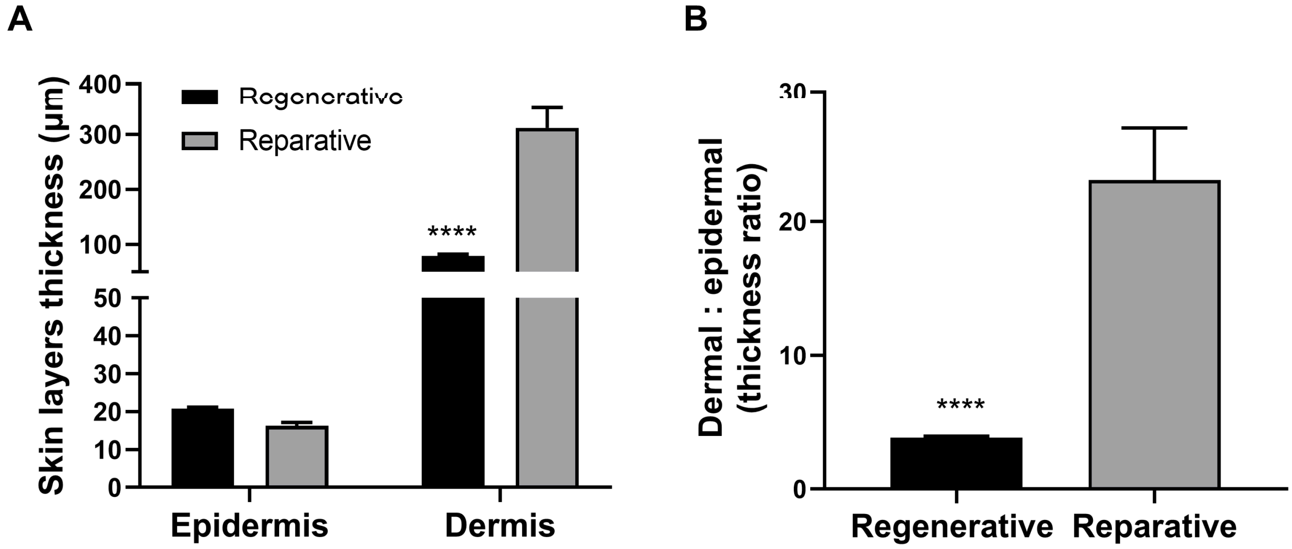

Supplementary Figure S1. Layer thickness of regenerative and reparative skin. (A) Epidermal and dermal thickness quantified from H&E staining. (B) Ration between dermal and epidermal thickness. Results are shown as Mean + SEM (N=4, 10 different measurements per sample) and analyzed using Mann Whitney and Mixed model comparison test, Tukey post-test. $p < 0.0001$.

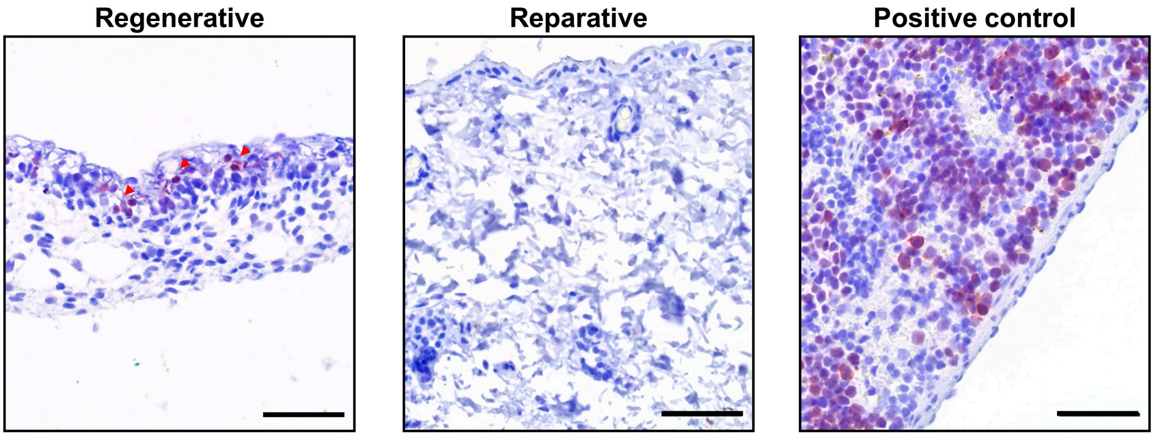

Supplementary Figure S2. Cell proliferation. Ki67 staining was performed to visualize mitotic cells, showing positive cells in the regenerative skin (red arrowheads) as well as mouse spleen (positive control). In contrast, none was detected in reparative skin. Scale bar represents 50 μm.
